# Optimisation of a Caprylic Acid-Based Protocol for IgG Purification from Baboon (*Papio anubis*) Serum

**DOI:** 10.3390/mps9010029

**Published:** 2026-02-22

**Authors:** Wathuto Ogopotse, Valentine Musabyimana, Pamela M. Khasandi, Dennis Kotti, Maina Ngotho, John M. Kagira, George O. Oluoch

**Affiliations:** 1 Kenya Snakebite Research and Intervention Centre, Kenya Institute of Primate Research, Ministry of Health, Nairobi P.O. Box 24481-00502, Kenya; vmusabyimana@primateresearch.org (V.M.); pkhasandi@primateresearch.org (P.M.K.); kottydennis@gmail.com (D.K.); george@primateresearch.org (G.O.O.); 2 Department of Molecular Biology and Biotechnology, Pan African University Institute for Basic Sciences, Technology and Innovation, Nairobi P.O. Box 62000-00200, Kenya; 3 Department of Animal Sciences, Jomo Kenyatta University of Agriculture & Technology, Nairobi P.O. Box 62000-00200, Kenya; mngotho@jkuat.ac.ke (M.N.); jkagira@jkuat.ac.ke (J.M.K.)

**Keywords:** antibody, purification, caprylic acid, optimisation, immunoglobulin G, baboon

## Abstract

Caprylic acid (CA) fractionation of serum is a simple and cost-effective method of producing high-quality immunoglobulins. While standardised procedures exist for CA purification of IgG for various animals, no published protocol exists for baboon IgG. This study aimed to optimise an efficient protocol for purifying IgG from baboon serum using CA through a stepwise one-factor-at-a-time (OFAT) approach. The effects of serum pH, CA concentration, stirring time and intensity, dialysis buffer, and lyophilisation were evaluated based on the protein content, with SDS-PAGE profiles and albumin–globulin ratios distinguishing IgG from residual albumin. Serum at pH 5.0 with 7% CA (*v*/*v*) produced the highest yield, minimising albumin while maximising IgG content. Lower pH (4.0–4.5) and CA (5–6%) reduced protein content, while a higher pH (5.5–6.0) and CA (8–15%) increased protein, but with elevated albumin and contaminants. Stirring serum vigorously at 1200 rpm for 60 min provided effective precipitation of non-IgG proteins. Lower intensities and shorter times resulted in higher albumin and residual proteins, while excessive stirring caused protein denaturation. Dialysis buffer composition had little impact, while lyophilisation significantly enhanced IgG concentration. The optimal protocol involved serum at pH 5.0, 7% CA (*v*/*v*), vigorous stirring (1200 rpm) for 60 min, and dialysis against sodium phosphate buffer (pH 7.4) followed by lyophilisation. The resulting IgG enrichment and purity were comparable to commercial-grade products. This study thus established optimal conditions for the purification of baboon IgG with CA, which could be used to support research in this animal model of immunology.

## 1. Introduction

Immunoglobulins (Igs), or antibodies, are glycoproteins produced by plasma cells, which make up about 20% of all plasma proteins [1]. When B cells are triggered by specific immunogens, they differentiate into plasma cells, which produce antibodies as part of the humoral immune response against pathogens and other foreign objects [1]. Studies have shown that the Ig sequences of baboons (*Papio anubis*) and humans (*Homo sapiens*) share a high level of genetic similarity, ranging from 87% to 90% [2,3]. This similarity has made *P. anubis* a valuable model for studying human pathophysiology associated with many diseases, including cardiovascular, respiratory, metabolic, and infectious diseases, as well as genetic, immunological, and reproductive disorders [4,5,6]. Baboons are also well-established models for the assessment of the humoral immune responses and pre-clinical efficacy of vaccine candidates developed against various human pathogens such as human immunodeficiency virus (HIV), Zika virus, Hepatitis B virus, *Schistosoma mansoni*, and *Bordetella pertussis* [7]. Furthermore, earlier studies have demonstrated the clinical applications of baboon-derived antibodies for the diagnosis of *Borrelia burgdorferi* infection [8] and as anti-idiotype antibodies against human carcinoembryonic antigen (CEA)-producing carcinomas [9].

Antibody purification from biological fluids is essential for various applications in research, diagnostics, and therapeutics [10]. Traditional methods like protein A, protein G, protein L (A/G/L) affinity chromatography, ion-exchange, and size-exclusion chromatography are effective but time-consuming and costly [10,11]. They are also prone to causing antibody aggregation, reducing antibody potency [10,11]. Non-chromatographic methods, such as precipitation, flocculation, crystallisation, and aqueous two-phase partitioning, offer simplicity and cost-effectiveness [12,13]. However, these methods have limitations in efficiency, scalability, and achieving consistent purity levels without contaminants [12,14].

Caprylic acid purification mitigates some of these challenges by incorporating mildly acidic conditions and hydrophobic interactions to selectively precipitate non-IgG proteins, leaving IgG in a soluble form [15,16]. This minimises the yield loss and the possibility of antibody aggregate formation, effectively reducing contaminants and resulting in a good safety profile [17]. Furthermore, CA precipitation has demonstrated complete clearance of model retroviruses and enveloped viruses, contributing to the overall virus safety of the process [15,18]. Several studies have highlighted the efficacy of CA purification in obtaining IgG fragments with high neutralisation activity, purity, and yield [19,20]. This method is quick, simple, and avoids the high costs and equipment requirements associated with traditional methods [19]. It is particularly advantageous in low-resource laboratories, where access to advanced technology is often limited [21].

Despite the widespread use of caprylic acid fractionation of serum for immunoglobulin purification in species such as horses, camels, sheep, and small laboratory animals, there is currently no systematic study optimising its application for baboon (*P. anubis*) serum. Studies involving non-human primates (NHPs) such as baboons are subject to stringent ethical regulations that, while essential for animal welfare, severely restrict blood sampling volume and frequency [22,23,24]. This necessitates protocols that are efficient at a small scale in order to preserve samples. Furthermore, IgG purification protocols developed for other species are not directly transferable to baboons due to differences in serum composition and immunoglobulin profiles [25]. These biological and ethical limitations, along with the high costs and logistical challenges of NHP studies, highlight a practical need for a simple, low-volume, and reproducible IgG purification protocol specifically adapted for baboon serum and suitable for laboratories operating under limited-resource conditions.

Various studies have identified key operational conditions for the CA-based purification of IgG, including pH, CA concentration, reaction time, stirring time, stirring intensity, and buffer components [15,16]. These factors have been examined for their impact on IgG yield and quality, as well as the removal of host cell proteins and high molecular weight aggregates for several species, including horses [17,26], sheep [20], and camels [16].

There are currently no studies demonstrating the specific conditions required for effective IgG purification from baboon serum. This study was carried out to optimise key parameters, including the caprylic acid concentration, serum pH, stirring time and intensity, dialysis buffer composition, and lyophilisation, to achieve the highest IgG yield and purity. The protein content of IgG, albumin concentration, albumin-to-immunoglobulin ratio, turbidity, and SDS-PAGE profiles were used as primary outcome measures.

To ensure ethical compliance with non-human primate sampling guidelines, the protocol was optimised using minimal serum volumes in a stepwise one-factor-at-a-time (OFAT) evaluation approach [27]. This ensured its applicability for laboratories operating under similar ethical and resource constraints. The aim of the present study was therefore to generate and optimise a simple and efficient protocol for the purification of IgG antibodies from baboon serum using caprylic acid.

## 2. Materials and Methods

### 2.1. Ethical Approval and Animal Welfare

This study was approved by the Institutional Scientific and Ethics Review Committee of the Kenya Institute of Primate Research (ISERC; Reference No. ISERC/05/19; approval date: 12 September 2019) and was conducted in accordance with institutional and international guidelines for the care and use of NHPs for scientific purposes. Twenty-four (24) healthy colony-born and colony-bred baboons of mixed sex, mixed age (1.5–4 years), and weight (4–10 kg) were used in this study. They were maintained at the regulated primate facility at the Kenya Institute of Primate Research (KIPRE), Animal Science Department, Animal Colony Facility, which is accredited by the Association for Assessment and Accreditation of Laboratory Animal Care International (AAALAC). The baboons were housed in well-maintained and adequately ventilated group enclosures, with sufficient enrichment devices. They were fed on nutrition-dense monkey cubes (Unga Farm Care East Africa, Ltd., Nairobi, Kenya) and supplemented with a variety of locally sourced vegetables and fruits. Drinking water was provided to the baboons ad libitum. The animals underwent routine health assessment prior to and during the study to monitor their hematologic, hepatic, and kidney statuses, all of which were in the normal ranges throughout the study period. Routine screening for tuberculosis and deworming was also carried out on a regular basis.

### 2.2. Collection of Blood Samples

During blood collection, animals were sedated by trained veterinary personnel using a chemical restraint consisting of xylazine (0.5 mg/mL; xylazine 2%, Agrar Holland BV, Soest, The Netherlands) and ketamine (10 mg/kg; ketamine 10%, Bremer Pharma GMBH, Warburg, Germany). Sampling was performed at 28-day intervals, and a maximum total of 9 mL of blood was collected per animal at each timepoint, in strict compliance with approved ethical limits. Of this volume, 6 mL were collected for serum processing and used in this study, while 3 mL were processed for plasma and stored. Blood samples for serum preparation were obtained by venipuncture of the femoral vein and collected into serum tubes (BD Vacutainer^®^ clot activator, silicone-coated interior, Becton Dickinson, Franklin Lakes, NJ, USA). Following clot formation, tubes were centrifuged at 3000 rpm (1850× *g*) for 5 min at 21 °C to obtain serum. The serum samples were aliquoted into sterile cryovials and stored at −20 °C until use.

#### Serum Preparation

Frozen serum samples were allowed to thaw at room temperature (20 to 25 °C) and pooled by mixing 0.5 mL of serum from each of the 24 baboons to a final volume of 12 mL. Due to limited serum samples, pooling was performed to generate sufficient volumes for protocol optimisation while reducing the biological variability between animals during the analysis of various parameters. A total number of seven independent serum pools were prepared and processed under identical conditions. Aliquots of 3 mL pooled serum were used to assess each experimental condition.

### 2.3. Antibody Purification with Caprylic Acid: Before Optimisation

Baboon serum fractionation with caprylic acid was carried out using the previously outlined procedure [28,29] with some modifications. Briefly, each pooled serum sample was heated at 56 °C for 15 min to deactivate complement activity, followed by centrifugation at 2090 rpm (900× *g*) for 10 min. The pooled serum was maintained at its original pH range of 7.5 to 8.0. Caprylic acid was then added slowly while constantly stirring the mixture at 800 rpm using a vortex (Vortex Stirrer 3000 rpm, Fisherbrand, Rodano, Italy) until a final concentration at 6% was reached. The mixture was stirred at room temperature for 30 min, followed by centrifugation at 11,000 rpm (12,840× *g*) for 45 min at 4 °C. The supernatant was then filtered through a 0.2 µm sterile syringe filter, and the pH of the supernatant was adjusted to 7.0 by the addition of 0.1 M sodium hydroxide (NaOH). Dialysis of the filtrate was performed against sodium citrate-buffered saline (SCS, pH 6.0) using Slide-A-Lyzer™ Dialysis Cassettes (3.5K MWCO) (Thermo Fisher Scientific, Rockford, IL, USA). Dialysis was performed for 1 h at room temperature, followed by a change of the buffer, then repeated overnight (~16 h) at 4 °C.

### 2.4. Optimisation and Standardisation of the Caprylic Acid Fractionation Protocol for Baboon Serum

To determine optimal conditions for the caprylic acid fractionation of baboon serum, the following parameters were evaluated: serum pH, caprylic acid concentration, duration of stirring, intensity of stirring, and composition of the dialysis buffer. Additionally, the effect of lyophilisation on the final IgG product was assessed. Each experimental setup was performed in triplicate to ensure reproducibility. Optimal conditions were defined as those producing the highest total protein concentration, while minimising residual albumin content, resulting in the lowest albumin-to-globulin ratio, low turbidity in the final product, and a clear IgG band on SDS-PAGE gels with minimal contaminating serum proteins. Each parameter was evaluated using a stepwise one-factor-at-a-time (OFAT) approach. All values were reported as mean ± SD of triplicate experiments (*n* = 3).

#### 2.4.1. Serum pH

Aliquots (3 mL) of pooled baboon serum were subjected to complement deactivation followed by centrifugation as described above. Acetic acid (1.0 M) was then used to adjust the pH of each sample to values of 4.0, 4.5, 5.0, 5.5, and 6.0. Caprylic acid was added to each aliquot up to a final concentration of 6% (*v*/*v*) with constant stirring at 800 rpm. The mixtures were stirred for 30 min at room temperature before they were centrifuged for 45 min at 11,000 rpm (12,840× *g*), and 4 °C. Supernatants were adjusted to pH 7.0 using 1.0 M NaOH and filtered. Dialysis was performed against sodium citrate-buffered saline (SCS, pH 6.0) for 1 h at room temperature, followed by a change of the buffer, then repeated overnight at 4 °C.

#### 2.4.2. Caprylic Acid Concentration

Aliquots (3 mL) of pooled baboon serum were subjected to complement deactivation, and the pH of each sample was adjusted to 5.0. Caprylic acid was then added slowly to each aliquot to achieve various concentrations of 5%, 6%, 7%, 8%, 9%, 10%, 12%, and 15% (*v*/*v*), while stirring continuously. After being stirred for 30 min at room temperature, all samples were subjected to centrifugation at 11,000 rpm (12,840× *g*) for 45 min at 4 °C. The pH of the supernatants was adjusted to 7.0 using 1.0 M NaOH, followed by filtration and then dialysis against SCS (pH 6.0) for 1 h at room temperature with a change of the buffer, then overnight at 4 °C.

#### 2.4.3. Stirring Intensity

Caprylic acid was added to sera (pH 5.0) to a final concentration of 7% (*v*/*v*) with constant stirring. The vortex was adjusted to four different intensities: mild stirring (200 rpm), moderate stirring (800 rpm), vigorous stirring (1200 rpm), and extremely vigorous stirring (1500 rpm), and the mixtures were stirred for 30 min at room temperature. The mixtures were then centrifuged at 11,000 rpm (12,840× *g*) for 45 min at 4 °C. The pH of the supernatants was adjusted to 7.0 by adding 1.0 M NaOH, followed by filtration. Dialysis was carried out against SCS (pH 6.0) for 1 h at room temperature with a change of the buffer, then repeated overnight at 4 °C.

#### 2.4.4. Stirring Time

Caprylic acid (7% *v*/*v* final concentration) is added to baboon serum (pH 5.0) slowly with constant stirring at 1200 rpm for different times: 30 min, 60 min, 90 min, and 120 min, respectively, at room temperature. The mixtures were centrifuged at 11,000 rpm (12,840× *g*) for 45 min at 4 °C. The pH of the supernatants was adjusted to 7.0 using 1.0 M NaOH, followed by filtration and dialysis against SCS (pH 6.0) for 1 h at room temperature with a change of the buffer, then overnight at 4 °C.

#### 2.4.5. Dialysis Buffer Composition

Caprylic acid (7% *v*/*v*) was added to sera (pH 5.0) with constant vigorous stirring (1200 rpm) for 60 min at room temperature. The mixtures were then centrifuged, and supernatants (pH 7.0) were filtered as described in Section 2.3 above. Dialysis was then carried out against different buffers: sodium citrate buffered saline (SCS, pH 6.0), sodium phosphate buffer (SPB, pH 7.4) and phosphate-buffered saline (PBS, pH 7.2). Each dialysis was carried out for 1 h at room temperature, followed by a change of the buffer, then overnight at 4 °C.

#### 2.4.6. Lyophilisation

Following the addition of CA (7% *v*/*v*) to sera (pH 5.0) with vigorous stirring (1200 rpm) for 60 min, the mixtures were centrifuged, and the supernatants (pH 7.0) were filtered as described in Section 2.3, then dialysed against an SPB (pH 7.4). This was followed by lyophilisation on a bench-top vacuum freeze-dryer (ScanVac CoolSafe freeze dryer, LaboGene, Allerød, Denmark) for 8 h. Lyophilised samples were then stored at 4 °C until reconstitution with PBS.

### 2.5. Purification of Baboon Serum with Caprylic Acid After Optimisation of the Protocol

Serum samples were pooled, and complement deactivation was performed, followed by centrifugation as described in Section 2.3. Caprylic acid (7% *v*/*v*) was added to the serum (pH 5.0) with constant vigorous stirring (1200 rpm). The mixture was left to shake for 60 min at room temperature and then centrifuged at 11,000 rpm (12,840× *g*) for 45 min. The pH of the supernatants was adjusted to 7.0 using 1.0 M NaOH, followed by filtration and dialysis against a sodium phosphate buffer (pH 7.4) using Slide-A-Lyzer™ Dialysis Cassettes (3.5K MWCO, Thermo Fisher Scientific, Rockford, IL, USA). Dialysis was performed for 1 h at room temperature (20–25 °C), followed by a change of the buffer, then repeated at 4 °C overnight. The final IgG sample was lyophilised and stored at 4 °C before reconstitution in PBS.

### 2.6. Characterisation of IgG Purified by Caprylic Acid Under Different Conditions

#### 2.6.1. Protein Quantification

The protein concentration of the samples was measured using a micro-volume spectrophotometer (BioDrop µLITE, Biochrom Ltd., Cambridge, UK). Concentrations were determined by the direct UV protein measurement using the inbuilt IgG mass extinction coefficient. Samples were measured in triplicate and presented as mean ± standard deviation. To ensure accurate equipment functionality, purified equine IgG (Bio-Rad Laboratories Inc., Hercules, CA, USA) was diluted in PBS (pH 7.2) to solutions of known concentrations (1000, 750, 500, and 250 μg/mL), which were used as standards and measured along with the experimental samples. The albumin concentration was determined by the bromocresol green method using an albumin colourimetric assay kit (catalog no: E-BC-K057-S, Elabscience Biotechnology Co., Ltd., Houston, TX, USA) according to the manufacturer’s instructions. Absorbance was measured in triplicate at 628 nm using a double-beam spectrophotometer (Model 6800, Jenway, Dunmow, UK).

#### 2.6.2. Sodium Dodecyl Sulphate–Polyacrylamide Gel Electrophoresis (SDS-PAGE)

All purified IgG preparations and IgG products used for comparisons were analysed by SDS-PAGE under reducing and non-reducing conditions using a 12% (*w*/*v*) separating gel, as described elsewhere [30]. Samples were mixed with 2× protein loading buffer (1.25 M Tris pH 6.8, 4% SDS, 20% glycerol, 2 mg bromophenol blue) containing β-mercaptoethanol (reduced) and without β-mercaptoethanol (non-reduced), then heated at 85 °C for 5 min and loaded on gels. Electrophoresis was performed at 200 V for 1 h at constant current (30 mA per gel). A protein ladder (PageRuler Plus Prestained Protein Ladder, 10–250 kDa, Thermo Fisher Scientific, Vilnius, Lithuania) was used as the molecular weight standard. The gels were stained with Coomassie blue R250, destained, and visualised using a gel documentation system (Uvidoc HD6, Uvitec Ltd., Cambridge, UK).

#### 2.6.3. Turbidity Measurement

Turbidity was evaluated by measuring the absorbance of each purified IgG sample in triplicate at 600 nm using a microplate reader (Model LT-4500, Labtech International Ltd., Uckfield, UK).

### 2.7. Comparison of Caprylic Acid-Purified Baboon IgG with Commercial IgG Products

The purified horse IgG used in this study was obtained from the manufacturer (Bio-Rad Laboratories Inc., Hercules, CA, USA). The whole IgG snake antivenom, EchiTAb-plus-ICP (Instituto Clodomiro Picado, Batch Number 5370114PALQ), was donated by the manufacturer. Baboon IgG purified from serum by caprylic acid fractionation was compared to purified horse IgG and commercial antivenom (EchiTAb-plus-ICP) in terms of total protein and albumin concentrations and albumin–immunoglobulin (albumin–globulin) ratios, as well as through assessment of the level of IgG enrichment, homogeneity, and purity by SDS-PAGE. Additionally, the turbidity of the purified IgG was evaluated in comparison with that of the commercial products. All data were presented as mean ± SD of triplicate experiments (*n* = 3).

### 2.8. Statistical Analyses

The raw data was entered into MS Excel (Microsoft Corporation, Redmond, WA, USA) spreadsheets and exported to GraphPad Prism version 9.5.0 for Windows (GraphPad Software, San Diego, CA, USA) for analysis. The differences between mean values for various IgG purification parameters (before and after optimisation) were analysed using ANOVA followed by Tukey’s multiple comparisons test, while comparisons with the control were made by ANOVA followed by Dunnett’s multiple comparisons test. A *p*-value cutoff of *p* < 0.05 was used to indicate statistical significance.

## 3. Results

### 3.1. Determination of the Optimal Conditions for Fractionation of Baboon Serum with Caprylic Acid

#### 3.1.1. pH Optimisation

**Figure 1:**
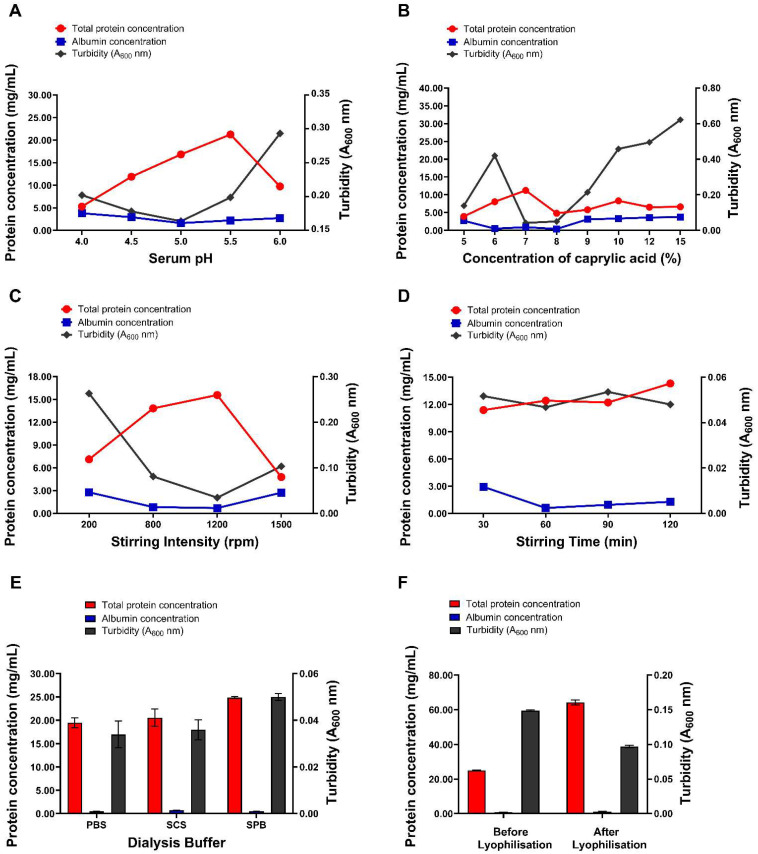
The effect of different conditions on caprylic acid fractionation of baboon antisera. Data is shown for total protein concentration (mg/mL), albumin concentration (mg/mL) and turbidity (absorbance at 600 nm) for (**A**) different serum pH values, (**B**) different concentrations of caprylic acid, (**C**) varying stirring intensities, (**D**) different stirring times, (**E**) different dialysis buffers, and (**F**) different lyophilisation conditions.

**Figure 2:**
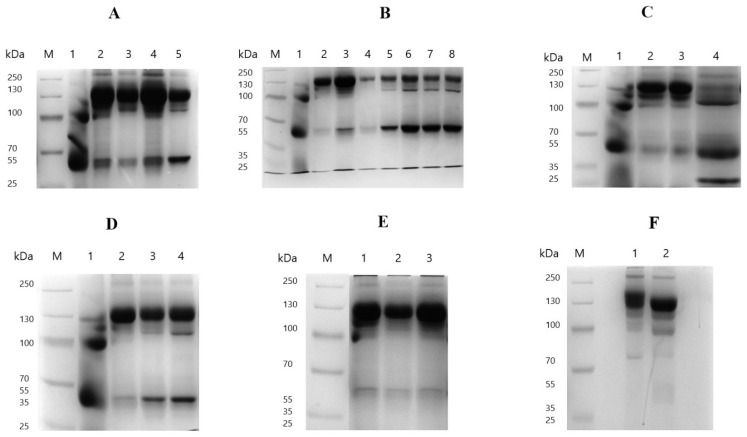
Non-reducing SDS-PAGE analysis of CA fractionation of baboon serum under various conditions. (**A**) Effect of serum pH: Lane M contains a protein marker, Lanes 1–5 represent pH values of 4.0, 4.5, 5.0, 5.5, and 6.0, respectively. (**B**) Effect of CA concentration: Lane M is the protein marker, Lanes 1–8 represent a CA concentration gradient (*v*/*v* %): 5%, 6%, 7%, 8%, 9%, 10%, 12%, and 15%, respectively. (**C**) Effect of stirring intensity: Lane M is the protein marker, Lanes 1–4 correspond to stirring intensities of 200 rpm, 800 rpm, 1200 rpm, and 1500 rpm, respectively. (**D**) Effect of stirring time: Lane M is the protein marker, Lanes 1–4 represent stirring times of 30 min, 60 min, 90 min, and 120 min, respectively. (**E**) Effect of dialysis buffers: Lane M is the protein marker, and lanes 1–3 represent different dialysis buffers: SCS, PBS, and SPB, respectively. (**F**) Effect of lyophilisation: Lane M is the protein marker, Lane 1 contains the non-lyophilised product, and Lane 2 contains the lyophilised product.

Caprylic acid fractionation of serum at the different pH values resulted in a significant increase in the protein concentration of purified fractions at pH 4.5 (11.88 ± 0.13 mg/mL), 5.0 (16.82 ± 0.09 mg/mL), 5.5 (21.24 ± 0.18 mg/mL) and 6.0 (9.77 ± 0.61 mg/mL) compared to the control (8.12 ± 0.51 mg/mL; *p* < 0.0001 for pH 4.5–5.5 and *p* = 0.0004 for pH 6.0) (Figure 1A; Table S1, Supplementary Materials). In contrast, at the lowest pH of 4.0, the protein concentration decreased significantly to 5.29 ± 0.09 mg/mL relative to the control (*p* < 0.0001). The albumin concentration was significantly lower at pH 5.0 (1.61 ± 0.46 mg/mL) and 5.5 (2.21 ± 0.17 mg/mL) compared to the control (3.42 ± 0.46 mg/mL; *p* = 0.0004 and *p* = 0.0089, respectively), whereas no significant differences were observed at pH 4.0, 4.5, or 6.0 (*p* > 0.05). The highest protein concentration was obtained after adjusting the pH of the serum to 5.5. However, adjustment to pH 5.0 produced significantly higher protein levels compared to pH 4.0, 4.5, and 6.0 (*p* < 0.0001), while also exhibiting the lowest albumin content, turbidity (0.16), and albumin/immunoglobulin ratio (0.11 ± 0.03), indicating higher IgG recovery. SDS-PAGE analysis of the resulting IgG purified at different pH values revealed prominent bands at approximately 150 kDa and 66 kDa, corresponding to whole IgG and albumin, respectively (Figure 2A; original gel images are provided in the Supplementary Materials). Smudging was observed in the well corresponding to the fraction purified at pH 4.0, indicating the presence of contaminants. Additionally, there were residual serum proteins detected in the purified fractions to varying degrees, represented by bands at approximately 90–100 kDa and 250 kDa. The fraction purified at pH 5.0 showed the most optimal fractionation, displaying a prominent band of whole IgG with minimal residual proteins and contaminants after purification. 

#### 3.1.2. Optimisation of Caprylic Acid Concentration

The results of baboon serum fractionation with different concentrations of CA are presented in Figure 1B, Table S2. The protein content of the purified fractions was significantly lower than that of the control (8.12 ± 0.51 mg/mL; *p* < 0.0001) at CA concentrations of 5% (3.92 ± 0.04 mg/mL), 8% (4.81 ± 0.12 mg/mL), 9% (5.79 ± 0.22 mg/mL), 12% (6.52 ± 0.37 mg/mL), and 15% (6.62 ± 0.09 mg/mL), while no significant differences were observed at 6% and 10% (*p* > 0.05). No significant decrease in albumin content was observed across most concentrations (5%, 9%, 10%, 12%, and 15%) compared to the control (*p* > 0.05), leading to the high turbidity of the fractions post-purification. However, albumin content was significantly reduced at 6% (0.50 ± 0.17 mg/mL), 7% (0.91 ± 0.30 mg/mL) and 8% (0.40 ± 0.17 mg/mL) compared with the control (3.42 ± 0.46 mg/mL; *p* < 0.0001), resulting in lower turbidity. The fraction purified with 7% CA yielded the highest protein content among all concentrations tested (11.23 ± 0.46 mg/mL), significantly surpassing the control (*p* < 0.0001). Additionally, its albumin content was significantly lower (0.91 ± 0.30 mg/mL) than that of the control (*p* < 0.0001, resulting in a very low albumin-to-immunoglobulin ratio and reduced turbidity (Table S2). Therefore, 7% CA was identified as the optimal concentration for baboon IgG purification. Further analysis by SDS-PAGE revealed that the fraction purified with 7% CA contained few contaminants, with a prominent IgG band and minimal serum proteins compared to other concentrations (Figure 2B). This further confirmed 7% CA as the optimal concentration for baboon serum fractionation.

#### 3.1.3. Optimisation of Stirring Intensity

The effects of different stirring intensities when mixing caprylic acid and serum during IgG purification are summarised in Figure 1C, Table S3. Mild (200 rpm) and extremely vigorous (1500 rpm) stirring significantly reduced protein content to 7.11 ± 0.17 mg/mL and 4.77 ± 0.15 mg/mL, respectively, compared to the control (8.12 ± 0.51 mg/mL; *p* = 0.0036 and *p* < 0.0001), without any notable differences in albumin levels (*p* > 0.05). In contrast, moderate (800 rpm) and vigorous (1200 rpm) stirring significantly increased protein concentrations to 13.81 ± 0.03 mg/mL and 15.57 ± 0.26 mg/mL, respectively, relative to the control (*p* < 0.0001 for both). These conditions also significantly reduced albumin levels to 0.82 ± 0.10 mg/mL and 0.70 ± 0.09 mg/mL, respectively, compared to the control (3.42 ± 0.46 mg/mL; *p* < 0.0001 for both). SDS-PAGE analysis of the purified fractions indicated prominent bands corresponding to intact IgG and albumin with some residual serum proteins (Figure 2C). However, fractions subjected to mild and extremely vigorous stirring displayed smudging, indicative of contaminants, as well as degraded IgG molecules, evidenced by distinct heavy (55 kDa) and light (25 kDa) chains. Vigorous stirring at 1200 rpm was found to be the optimal stirring intensity, yielding the highest protein content of IgG (15.57 ± 0.26 mg/mL) along with the lowest albumin concentration (0.70 ± 0.09 mg/mL), albumin–immunoglobulin ratio (0.05 ± 0.01), and turbidity (0.03) (Table S3). Furthermore, SDS-PAGE analysis revealed prominent IgG bands with minimal contaminants in 1200 rpm fractions, further highlighting it as the optimal stirring intensity for baboon serum fractionation (Figure 2C).

#### 3.1.4. Optimisation of Stirring Time

Following the addition of caprylic acid to sera, the mixtures were stirred vigorously (1200 rpm) for different periods to evaluate the effect of stirring time on IgG purification. All stirring times resulted in significantly higher protein concentrations compared to the control (*p* < 0.0001) (Figure 1D, Table S4). The albumin content was significantly reduced when stirring was performed for 60 min (0.60 ± 0.03 mg/mL), 90 min (0.94 ± 0.00 mg/mL), and 120 min (1.27 ± 0.11 mg/mL) compared to the control (3.42 ± 0.46 mg/mL; *p* < 0.0001), whereas no significant reduction was observed at 30 min (2.92 ± 0.35 mg/mL, *p* = 0.1220). SDS-PAGE analysis of purified fractions revealed distinct bands corresponding to intact IgG and albumin, along with residual serum proteins (Figure 2D). The fraction stirred for 30 min exhibited a higher level of residual serum proteins and contaminants, as indicated by smearing in the corresponding gel lane. Overall, the results showed that stirring for 60 min produced the best results, yielding the highest protein content (12.41 ± 0.01 mg/mL), the lowest albumin concentration (0.60 ± 0.03 mg/mL), and albumin–immunoglobulin ratio (0.05 ± 0.00) as well as turbidity (0.05) (Table S4). This was further corroborated by the electrophoretic profile, which showed a high level of IgG purity and enrichment in the fraction stirred for 60 min, confirming this as the optimal stirring time.

#### 3.1.5. Optimisation of Dialysis Buffer

The effect of using three different dialysis buffers on baboon IgG purification is illustrated in Figure 1E, Table S5. Fractions dialysed against all the buffers exhibited significantly higher protein concentrations and significantly lower albumin concentrations compared to the control (*p* < 0.0001). While no significant differences were observed in albumin concentrations across the different buffers (*p* > 0.05), the fraction dialysed against SPB exhibited a slightly higher total protein concentration (24.87 ± 0.24 mg/mL) than both PBS (19.46 ± 1.06 mg/mL, *p* = 0.0014) and SCS (20.54 ± 1.85 mg/mL, *p* = 0.0058). SDS-PAGE analysis of purified fractions revealed prominent bands at approximately 150 kDa and 66 kDa representing intact IgG and albumin, respectively, with minimal residual serum proteins in all fractions (Figure 2E). Dialysis against SPB yielded the best results, providing the highest protein content, the lowest albumin concentration (0.52 ± 0.04 mg/mL), reduced turbidity (0.05), and the lowest albumin–immunoglobulin ratio (0.00), indicating enhanced IgG enrichment. These findings were further supported by the electrophoretic profiles, which demonstrated great purity, homogeneity, and fewer contaminating proteins in the fraction dialysed against SPB (Figure 2E).

#### 3.1.6. Effect of Lyophilisation on Final IgG Product

To further enhance the concentration of the purified IgG after optimising other parameters, lyophilisation was performed, and its effect on the final product is presented in Figure 1F, Table S6. The results show that lyophilisation enabled reconstitution of the purified IgG to a markedly higher concentration, resulting in a significantly greater total protein content (64.23 ± 1.46 mg/mL) compared to the non-lyophilised fraction (24.87 ± 0.24 mg/mL; *p* < 0.0001), as well as the control (8.12 ± 0.51 mg/mL; *p* < 0.0001). Although the albumin concentration showed a slight increase after lyophilisation (1.09 ± 0.20 mg/mL) relative to the non-lyophilised fraction (0.74 ± 0.03 mg/mL; *p* = 0.9175), this difference was not statistically significant. Consequently, the albumin–immunoglobulin ratio decreased from 0.03 to 0.02, reflecting an enhanced purification profile and a higher IgG content in the reconstituted lyophilised product. Furthermore, the turbidity of the reconstituted lyophilised fraction was significantly reduced to 0.10 from 0.15 (non-lyophilised fraction), indicating a clearer solution with less aggregation or insoluble particles. SDS-PAGE analysis of the purified fractions revealed prominent bands corresponding to intact IgG, with only faint bands attributable to albumin. Both fractions displayed minimal residual serum proteins and contaminants (Figure 2F). Overall, the lyophilised product demonstrated superior IgG enrichment and purity, with reduced contamination compared to the non-lyophilised product.

### 3.2. Analysis of Purified Baboon IgG Before and After Optimisation of the Caprylic Acid Fractionation Method

**Figure 3:**
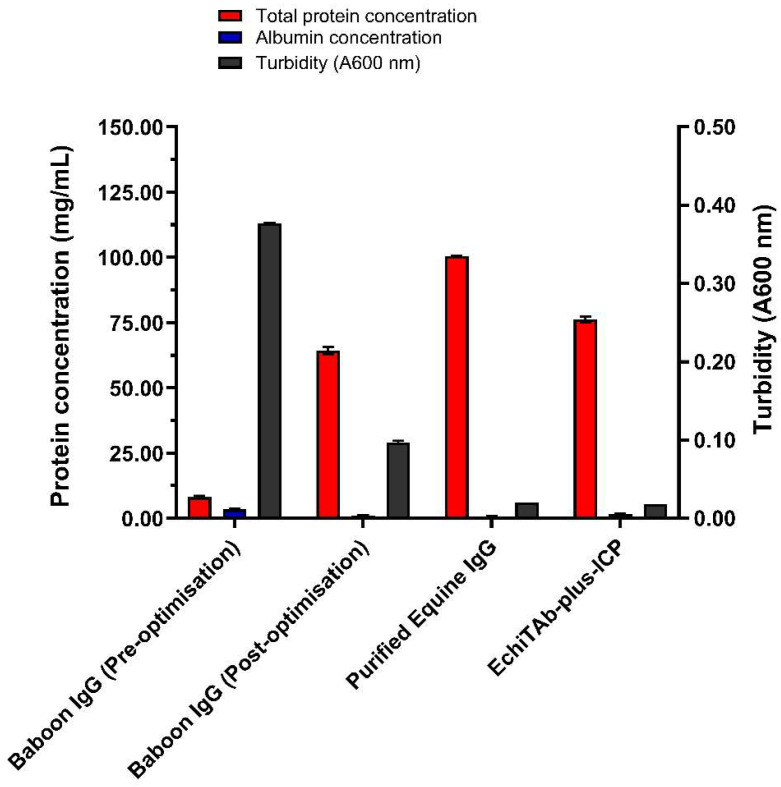
Comparison of caprylic acid-purified baboon IgG before and after optimisation and with commercial IgG products.

**Figure 4:**
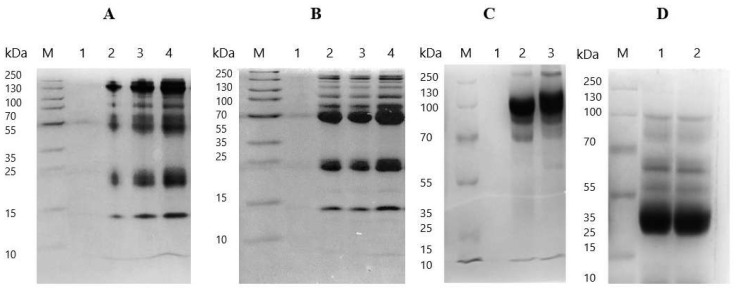
SDS-PAGE gel visualising protein profiles of baboon IgG before and after optimisation of the purification protocol, diluted to 1:100. (**A**) Non-reduced protein profile of baboon IgG before optimisation: Lane M: protein ladder; Lane 1: empty, Lanes 2–4: purified IgG at volumes of 5, 10, and 20 µL, respectively. (**B**) Reduced protein profile before optimisation: Lane M: protein ladder; Lane 1: empty, Lanes 2–4: purified IgG at volumes of 5, 10, and 20 µL, respectively. (**C**) Non-reduced protein profile after optimisation: Lane M: protein ladder; Lanes 2–3: purified IgG at volumes of 10 and 20 µL, respectively. (**D**) Reduced protein profile after optimisation: Lane M: protein ladder; Lanes 1–2: purified IgG at volumes of 10 and 20 µL, respectively.

A comparison of the caprylic acid-purified baboon IgG obtained before and after optimisation of the various purification conditions shows that the optimisation steps employed significantly improved the final product in terms of IgG enrichment, purity, and homogeneity (Figure 3, Table S7). The optimised protocol incorporated the use of 7% CA, maintaining the serum at pH 5.0, stirring at 1200 rpm for 60 min, and dialysis against the sodium phosphate buffer (SPB, pH 7.4), followed by lyophilisation of the final product. The results indicate that purification using this protocol exhibited a significantly higher total protein content (64.23 ± 1.46 mg/mL) and a lower albumin concentration (1.09 ± 0.20 mg/mL) compared to the product obtained before optimisation (27.93 ± 1.21 mg/mL and 4.23 ± 0.80 mg/mL, respectively; *p* < 0.0001 for both). Additionally, the albumin–immunoglobulin ratio (0.02 ± 0.00) and turbidity (0.10) of the final purified IgG fraction were much lower compared to before optimisation (0.18 ± 0.03 and 0.15 respectively). SDS-PAGE analysis provided further evidence of the improved quality of the optimised fraction, showing distinct and prominent bands corresponding to intact IgG at ~150 kDa under non-reducing conditions, and heavy (~55 kDa) and light (~25 kDa) chains under reducing conditions (Figure 4). Minimal residual serum proteins were observed in the optimised fraction (Figure 4C,D), whereas the pre-optimisation fraction exhibited notable contaminating bands, indicative of lower purity (Figure 4A,B). Overall, the optimised protocol yielded a product with higher purity, reduced contamination, and improved clarity, highlighting the effectiveness of the various modifications in improving IgG purification.

### 3.3. Comparison of Caprylic Acid Purified Baboon IgG with Commercial IgG Products

**Figure 5:**
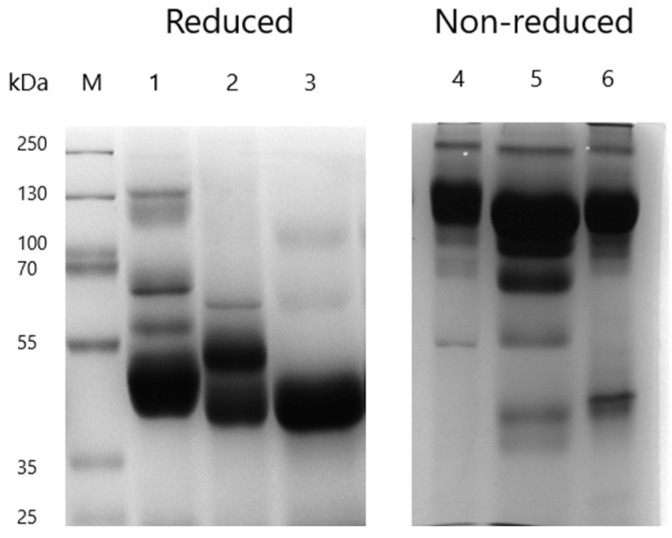
SDS-PAGE gels visualising protein profiles of caprylic acid-purified baboon IgG compared to commercial IgG products. Lane M: protein ladder; Lane 1: reduced profile of baboon IgG; Lane 2: reduced profile of equine IgG; Lane 3: reduced profile of EchiTAb-plus-ICP antivenom; Lane 4: non-reduced profile of EchiTAb-plus-ICP; Lane 5: non-reduced profile of baboon IgG; Lane 6: non-reduced profile of equine IgG.

The caprylic acid purified baboon IgG was compared to whole IgG snake antivenom (EchiTAb-plus-ICP) and purified equine IgG based on protein concentrations, albumin–immunoglobulin ratios, turbidity, and IgG enrichment, as assessed by SDS-PAGE (Figure 3, Table S8). Baboon IgG exhibited a high protein concentration of 64.23 ± 1.46 mg/mL, although it was significantly lower than that of EchiTAb-plus-ICP (76.17 ± 1.16 mg/mL; *p* < 0.0001) and equine IgG (100.46 ± 0.17 mg/mL; *p* < 0.0001). There was no significant difference in albumin content between the three products (*p* > 0.05). The baboon IgG demonstrated a higher albumin–globulin ratio (0.02 ± 0.00) compared to equine IgG (0.01 ± 0.00), but the ratio was similar to that of EchiTAb-Plus-ICP. While the purified baboon IgG showed reduced turbidity compared to its pre-optimised form and formed a clear solution after reconstitution, its turbidity (0.10) was much higher than that of the commercial IgG products (both 0.02). SDS-PAGE analysis of the products revealed prominent bands for intact IgG and faint bands for residual albumin under non-reducing conditions. Under reducing conditions, distinct bands corresponding to the heavy (~55 kDa) and light (~25 kDa) chains were observed (Figure 5). All products displayed minimal residual proteins and contaminants, although baboon IgG had a slightly higher level of residual serum proteins. Overall, the CA-purified baboon IgG demonstrated comparable IgG enrichment to commercial products, emphasising the effectiveness of the optimised protocol as a practical approach for IgG purification from baboon serum.

## 4. Discussion

In this study, IgG antibodies were purified from baboon serum using caprylic acid (CA) fractionation. This method has consistently yielded high-quality IgGs from the serum and plasma of various animals, including camels, horses, and sheep [16,19,20]. It is simple and cost-effective and does not require a significant investment in equipment. Due to its affordability and efficiency, this method is recommended for therapeutic antibody production in resource-limited settings, particularly in Sub-Saharan Africa (SSA), where access to advanced technologies is often limited [19,21].

We optimised baboon IgG purification using caprylic acid by evaluating the effect of key parameters, including CA concentration, serum pH, stirring conditions, dialysis buffer composition, and lyophilisation. Previous research highlights the importance of optimising these parameters to enhance the purification efficiency based on the specific properties of serum and the quality requirements of the IgG [31,32]. A stepwise OFAT strategy was used to optimise the various parameters that enabled thorough assessment of single variables while conserving sample volume [27], thus ensuring strict compliance with non-human primate ethical guidelines on sampling volume and frequency.

The concentration of CA and the pH of serum were found to be critical factors in the purification process. Our findings showed that 7% (*v*/*v*) CA, added to serum adjusted to pH 5.0, produced the best results, yielding an IgG of high purity while minimising residual albumin and other non-IgG proteins. Trapp et al. [18] reported that CA disrupts protein–protein and protein–solvent interactions, promoting the selective precipitation of unwanted proteins. Morais and Massaldi [33] further proposed that CA binds to specific sites on proteins, inducing partial unfolding and enhancing hydrophobic interactions, ultimately leading to precipitation. These studies highlight the importance of carefully controlling CA concentration, as excessive levels may cause over-precipitation and loss of IgG, while insufficient concentrations may fail to effectively remove unwanted proteins and impurities [18,33].

Other studies have reported various optimal CA concentrations and serum pH depending on the animal source. For instance, Nudel et al. and Miranda-Cruz et al. optimised CA at 3% for equine plasma at a pH of 4.9 and 4.5 respectively [17,26], while Shawki and colleagues [16] found 8% CA at pH 5.5 ideal for camel IgG purification. Concentrations of 5% and 6% CA at a serum pH of 4.5 have been reported as optimal for ovine IgG purification [29,34].

Serum pH plays a crucial role in IgG purification, affecting protein charge and solubility [18,32]. This study identified pH 5.0 as optimal for selectively precipitating non-IgG proteins while maintaining IgG solubility, resulting in a high yield and purity. This aligns with previous findings that at slightly acidic pH, the hydrophobic tail of CA prevails, causing acidic proteins to precipitate, while basic molecules like IgG counteract the hydrophobicity and remain soluble, preserving their stability [15]. Lower pH led to reduced yield and purity, consistent with studies showing that it destabilises IgG by disrupting ionic bonds, leading to denaturation and fragmentation [35,36]. Our findings also demonstrated that above the optimal pH, there was a low IgG content. This is reported in previous research, indicating that higher pH increases the negative charge in IgG molecules, hindering non-IgG protein precipitation, and reducing yield [15,35].

The stirring time and intensity when mixing CA with serum also affect the efficiency of purification. We found that stirring vigorously for 60 min at 1200 rpm was optimal for achieving the desired purity and yield in this study. This aligns with optimisations of stirring conditions for horse serum, as demonstrated by Rojas et al. [37], Miranda-Cruz et al. [26], and Nudel et al. [17]. These studies have demonstrated that vigorous stirring ensures thorough mixing of CA with serum, thus promoting consistent precipitation of non-IgG proteins. However, Shawki and co-workers [16] reported that, for camel serum, stirring at 450 rpm for 90 min provides optimal results, indicating that stirring conditions may vary depending on species.

Our results also showed that excessive stirring for long periods can lead to protein denaturation, an effect that can compromise both the yield and quality of purified IgG. Prior research indicates that overly vigorous or prolonged stirring introduces high shear forces, which can disrupt protein structure and stability, causing IgG to lose its functional conformation [38,39]. Furthermore, it has been demonstrated that inadequate stirring can result in incomplete mixing, leading to an inefficient separation of IgG from other proteins [37]. Therefore, optimising stirring intensity and duration is crucial to maximising the IgG concentration and purity while minimising denaturation.

After CA precipitation, dialysis is typically employed to remove any remaining CA and other impurities from the IgG fraction [34,40]. Our study showed that the composition of the dialysis buffer had minimal impact on the final concentration and purity of the purified IgG, which is consistent with previous reports [32]. This efficacy across different buffer systems simplifies the purification process, making it more adaptable to various laboratory settings without compromising the quality of the final product [32].

Lyophilisation is a method of choice for the long-term storage of antibodies, which enhances their physicochemical properties and stability, extends their shelf life, and reduces aggregation, albeit at added cost [41]. This study confirmed that lyophilisation increases the total protein yield by enabling an effective concentration of the IgG and improves the relative purity of the final product, aligning with previous findings [41,42]. Comparative analysis of the CA-purified baboon IgG with commercial IgG products, including EchiTAb-plus-ICP snake antivenom and purified equine IgG, demonstrated the effectiveness of our protocol in enriching IgG under defined experimental conditions. However, the comparisons were only limited to physicochemical measurements and SDS-PAGE profiling. Consequently, no associations can be drawn regarding the antigen-binding capacity or the biological and functional activity of the purified IgG generated in this study. Future studies building on this protocol should incorporate functional and immunochemical assays, such as antigen-binding immunoassays and in vitro or in vivo neutralisation tests, to further characterise IgG performance. Additionally, for clinical-grade production, additional downstream polishing steps could be integrated following CA fractionation, together with comprehensive safety and quality assessments, in order achieve purity and quality levels consistent with regulatory standards.

## 5. Conclusions

In this study, a caprylic acid-based protocol was optimised for the purification of IgG from baboon (*P. anubis*) serum, yielding IgG of high concentration, purity, and homogeneity. Key parameters, including CA concentration, serum pH, stirring conditions, dialysis buffers, and lyophilisation conditions, were optimised to maximise IgG recovery and purity while reducing albumin and other serum contaminants. Although exploratory in nature, this protocol holds great potential for applications in research involving baboon-derived antibodies. Furthermore, this simple and cost-effective method provides a robust and practical research-grade approach for the preparation of baboon IgG and is particularly suitable for laboratories operating under ethical, logistical, and resource constraints. However, while the protocol offers a strong foundation for downstream applications, additional functional validation and purification steps would be required for therapeutic and large-scale use.

## Data Availability

The authors confirm that the data supporting the findings of this study are available within the article and/or its Supplementary Materials. Further enquiries may be directed to the corresponding author.

## Supplementary Materials

The following supporting information can be downloaded at: https://www.mdpi.com/article/10.3390/mps9010029/s1, Table S1: The effect of different serum pH values on caprylic acid purification of baboon IgG; Table S2: The effect of different caprylic acid concentrations on the purification of baboon IgG; Table S3: The effect of different stirring intensities of sera on caprylic acid purification of baboon IgG; Table S4: The effect of different stirring times on caprylic acid fractionation of baboon antisera; Table S5: The effect of different dialysis buffers on caprylic acid fractionation of baboon antisera; Table S6: The effect of lyophilisation on the final IgG product purified using caprylic acid; Table S7: Comparison of the purified baboon IgG before and after optimisation of the caprylic acid purification protocol; Table S8: Comparison of caprylic acid purified baboon IgG with commercial IgG products.

## Abbreviations

The following abbreviations are used in this manuscript:

CA	Caprylic acid
Igs	Immunoglobulins
OFAT	One factor at a time
HIV	Human immunodeficiency virus
CEA	Carcinoembryonic antigen
USA	United States of America
UK	United Kingdom
IgG	Immunoglobulin G
SDS-PAGE	Sodium dodecyl sulfate–polyacrylamide gel electrophoresis
*v*/*v*	Volume per volume
NaOH	Sodium hydroxide
MWCO	Molecular weight cut-off
SCS	Sodium citrate-buffered saline
PBS	Phosphate-buffered saline
SPB	Sodium phosphate buffer
ANOVA	Analysis of variance
PAUSTI	Pan-African University Institute for Basic Sciences, Technology and Innovation
K-SRIC	Kenya Snakebite Research and Intervention Centre
KIPRE	Kenya Institute of Primate Research

## References

[1] Jamal Z., Patel P., Ramphul K. (2023). Immunoglobulin. StatPearls [Internet].

[2] Attanasio R., Jayashankar L., Engleman C.N., Scinicariello F. (2002). Baboon Immunoglobulin Constant Region Heavy Chains: Identification of Four IGHG Genes. Immunogenetics.

[3] Scinicariello F., Jayashankar L., Attanasio R. (2002). Baboon Immunoglobulin Variable Region Heavy Chains: Identification of Genes Homologous to Members of the Human IGHV1-IGHV7 Subgroups. Immunogenetics.

[4] Tarantal A.F., Noctor S.C., Hartigan-O’connor D.J. (2022). Nonhuman Primates in Translational Research. Annu. Rev. Anim. Biosci..

[5] Cox L.A., Comuzzie A.G., Havill L.M., Karere G.M., Spradling K.D., Mahaney M.C., Nathanielsz P.W., Nicolella D.P., Shade R.E., Voruganti S. (2013). Baboons as a Model to Study Genetics and Epigenetics of Human Disease. ILAR J..

[6] Mulholland M.M., Nehete B.P., DeLise A., Achorn A.M., Pytka L.M., Nehete P.N. (2024). Age-Associated Alterations in Immune and Inflammatory Responses in Captive Olive Baboons (*Papio anubis*). Front. Aging.

[7] Rivera-Hernandez T., Carnathan D.G., Moyle P.M., Toth I., West N.P., Young P.L., Silvestri G., Walker M.J. (2014). The Contribution of Non-Human Primate Models to the Development of Human Vaccines. Discov. Med..

[8] Hefty P.S., Brooks C.S., Jett A.M., White G.L., Wikel S.K., Kennedy R.C., Akins D.R. (2002). OspE-Related, OspF-Related, and Elp Lipoproteins Are Immunogenic in Baboons Experimentally Infected with *Borrelia burgdorferi* and in Human Lyme Disease Patients. J. Clin. Microbiol..

[9] Losman M.J., Monestier M., Hansen H.J., Goldenberg D.M. (1990). Baboon anti-idiotype antibodies mimic a carcinoembryonic antigen epitope. Int. J. Cancer.

[10] Arakawa T., Tomioka Y., Nakagawa M., Sakuma C., Kurosawa Y., Ejima D., Tsumoto K., Akuta T. (2023). Non-Affinity Purification of Antibodies. Antibodies.

[11] Milne J.J. (2017). Scale-Up of Protein Purification: Downstream Processing Issues. Methods Mol. Biol..

[12] Mukhtar H., Majeed Z., Naseer B., Iftikhar Shah F. (2021). Efficiency of Purification Techniques of Human IgG—A Review. J. Bacteriol. Mycol. Open Access.

[13] O’Kennedy R., Murphy C., Devine T. (2016). Technology Advancements in Antibody Purification. Antib. Technol. J..

[14] Van Alstine J.M., Jagschies G., Łacki K.M., Jagschies G., Lindskog E., Łącki K., Galliher P. (2018). Alternative Separation Methods: Flocculation and Precipitation. Biopharmaceutical Processing: Development, Design, and Implementation of Manufacturing Processes.

[15] Brodsky Y., Zhang C., Yigzaw Y., Vedantham G. (2012). Caprylic Acid Precipitation Method for Impurity Reduction: An Alternative to Conventional Chromatography for Monoclonal Antibody Purification. Biotechnol. Bioeng..

[16] Shawki A., Abd El-Baky N., Ahmed M., Linjawi M.H., Aljaddawi A.A., Redwan E.M. (2017). Simple Protocol for Immunoglobulin G Purification from Camel “*Camelus dromedarius*” Serum. Open Life Sci..

[17] Nudel B.C., Perdoménico C., Iácono R., Cascone O. (2012). Optimization by Factorial Analysis of Caprylic Acid Precipitation of Non-Immunoglobulins from Hyperimmune Equine Plasma for Antivenom Preparation. Toxicon.

[18] Trapp A., Faude A., Hörold N., Schubert S., Faust S., Grob T., Schmidt S. (2018). Multiple Functions of Caprylic Acid-Induced Impurity Precipitation for Process Intensification in Monoclonal Antibody Purification. J. Biotechnol..

[19] Gutiérrez J.M., Rojas E., Quesada L., León G., Núñez J., Laing G.D., Sasa M., Renjifo J.M., Nasidi A., Warrell D.A. (2005). Pan-African Polyspecific Antivenom Produced by Caprylic Acid Purification of Horse IgG: An Alternative to the Antivenom Crisis in Africa. Trans. R. Soc. Trop. Med. Hyg..

[20] Al-Abdulla I., Casewell N.R., Landon J. (2014). Single-Reagent One-Step Procedures for the Purification of Ovine IgG, F(Ab′)2 and Fab Antivenoms by Caprylic Acid. J. Immunol. Methods.

[21] El-Ekiaby M., Vargas M., Sayed M., Gorgy G., Goubran H., Radosevic M., Burnouf T. (2015). Minipool Caprylic Acid Fractionation of Plasma Using Disposable Equipment: A Practical Method to Enhance Immunoglobulin Supply in Developing Countries. PLoS Neglected Trop. Dis..

[22] European Commission, Scientific Committee on Animal Health and Animal Welfare (2002). The Welfare of Non-Human Primates Used in Research.

[23] Carvalho C., Gaspar A., Knight A., Vicente L. (2019). Ethical and Scientific Pitfalls Concerning Laboratory Research with Non-Human Primates, and Possible Solutions. Animals.

[24] Buckley L.A., Chapman K., Burns-Naas L.A., Todd M.D., Martin P.L., Lansita J.A. (2011). Considerations Regarding Nonhuman Primate Use in Safety Assessment of Biopharmaceuticals. Int. J. Toxicol..

[25] Bjornson-Hooper Z.B., Fragiadakis G.K., Spitzer M.H., Chen H., Madhireddy D., Hu K., Lundsten K., McIlwain D.R., Nolan G.P. (2022). A Comprehensive Atlas of Immunological Differences Between Humans, Mice, and Non-Human Primates. Front. Immunol..

[26] Miranda-Cruz A.R., Sánchez-Artigas R., Otero-Alfaro O., Góngora-Amores W., Cobos-Valdes D., Goya-Batista Y., Balboa-González J., Pérez-Martin O. (2014). Using the caprylic acid in obtaining the horse immunoglobulin anti tetanus toxin. VacciMonitor.

[27] Yu I.T. (2024). Factor Screening with Modified One-Factor-at-a-Time Experiments. Commun. Stat. Simul. Comput..

[28] Guidolin F.R., Caricati C.P., Marcelino J.R., da Silva W.D. (2016). Development of Equine IgG Antivenoms against Major Snake Groups in Mozambique. PLoS Neglected Trop. Dis..

[29] Alomran N., Alsolaiss J., Albulescu L.O., Crittenden E., Harrison R.A., Ainsworth S., Casewell N.R. (2022). Pathology-specific experimental antivenoms for haemotoxic snakebite: The impact of immunogen diversity on the in vitro cross-reactivity and in vivo neutralisation of geographically diverse snake venoms. PLoS Neglected Trop. Dis..

[30] Blancher C., Jones A. (2001). SDS -PAGE and Western Blotting Techniques. Methods Mol. Med..

[31] Taherian A., Fazilati M., Moghadam A.T., Tebyanian H. (2018). Optimization of Purification Procedure for Horse F(Ab´)2 Antivenom against *Androctonus crassicauda* (Scorpion) Venom. Trop. J. Pharm. Res..

[32] Zheng J., Wang L., Twarowska B., Laino S., Sparks C., Smith T., Russell R., Wang M. (2015). Caprylic Acid-Induced Impurity Precipitation from Protein A Capture Column Elution Pool to Enable a Two-Chromatography-Step Process for Monoclonal Antibody Purification. Biotechnol. Prog..

[33] Morais V., Massaldi H. (2012). A Model Mechanism for Protein Precipitation by Caprylic Acid: Application to Plasma Purification. Biotechnol. Appl. Biochem..

[34] Redwan E.R.M., Fahmy A., EL Hanafy A., EL-Baky N.A., Sallam S.M.A. (2009). Ovine Anti-Rabies Antibody Production and Evaluation. Comp. Immunol. Microbiol. Infect. Dis..

[35] Latypov R.F., Hogan S., Lau H., Gadgil H., Liu D. (2012). Elucidation of Acid-Induced Unfolding and Aggregation of Human Immunoglobulin IgG1 and IgG2 Fc. J. Biol. Chem..

[36] Lopez E., Scott N.E., Wines B.D., Hogarth P.M., Wheatley A.K., Kent S.J., Chung A.W. (2019). Low PH Exposure During Immunoglobulin G Purification Methods Results in Aggregates That Avidly Bind Fcγ Receptors: Implications for Measuring Fc Dependent Antibody Functions. Front. Immunol..

[37] Rojas G., Jiménez J., Gutiérrez J. (1994). Caprylic acid fractionation of hyperimmune horse plasma: Description of a simple procedure for antivenom production. Toxicon.

[38] Eppler A., Weigandt M., Hanefeld A., Bunjes H. (2010). Relevant Shaking Stress Conditions for Antibody Preformulation Development. Eur. J. Pharm. Biopharm..

[39] Kiese S., Papppenberger A., Friess W., Mahler H.C. (2008). Shaken, Not Stirred: Mechanical Stress Testing of an IgG1 Antibody. J. Pharm. Sci..

[40] Raweerith R., Ratanabanangkoon K. (2003). Fractionation of Equine Antivenom Using Caprylic Acid Precipitation in Combination with Cationic Ion-Exchange Chromatography. J. Immunol. Methods.

[41] Harris R.J., Shire R.J., Winter C. (2004). Commercial Manufacturing Scale Formulation and Analytical Characterization of Therapeutic Recombinant Antibodies. Drug Dev. Res..

[42] Sarciaux J.M., Mansour S., Hageman M.J., Nail S.L. (1999). Effects of Buffer Composition and Processing Conditions on Aggregation of Bovine IgG during Freeze-Drying. J. Pharm. Sci..

