# Supplementary material for: Optimisation of a Caprylic Acid-Based Protocol for IgG Purification from Baboon (Papio anubis) Serum
Source: Methods Protoc. 2026 Feb 22;9(1):29. doi: 10.3390/mps9010029 (PMC12943085; doi:10.3390/mps9010029)
Supplement: Supplementary file 1 [file mps-09-00029-s001.zip › Supplementary Information.pdf]

Supplementary Information

Optimisation of a Caprylic Acid–Based Protocol for IgG Purification from Baboon (*Papio anubis*) Serum

Wathuto Ogopotse[1, 2, *], Valentine Musabyimana[1], Pamela M. Khasandi[1], Dennis Kotti[1], Maina Ngotho[3], John M. Kagira[3], George O. Oluoch[1]

[1] Kenya Snakebite Research and Intervention Centre, Kenya Institute of Primate Research, Ministry of Health, P.O. Box 24481-00502, Karen, Nairobi, Kenya; vmusabyimana@primateresearch.org (V.M), pkhasandi@primateresearch.org (P.M.K), kottydennis@gmail.com (D.K), george@primateresearch.org (G.O.O)

[2] Department of Molecular Biology and Biotechnology, Pan-African University Institute of Basic Sciences, Technology and Innovation, P.O. Box 62000-00200, Nairobi, Kenya

[3]Department of Animal Sciences, Jomo Kenyatta University of Agriculture & Technology, P.O. Box 62000-00200 Nairobi, Kenya; mngotho@jkuat.ac.ke (M.N), jkagira@jkuat.ac.ke (J.M.K)

* Correspondence: wathutoogopotse@gmail.com

Table S1. The effect of different serum pH values on caprylic acid purification of baboon IgG.

pH of serum	Protein concentration (mg/mL)	Albumin concentration (mg/mL)	Albumin/ Globulin ratio	Turbidity (A600 nm)
4.0	5.29 ± 0.09*	3.79 ± 0.15	2.56 ± 0.35	0.20
4.5	11.88 ± 0.13*	2.92 ± 0.17	0.33 ± 0.03	0.18
5.0	16.82 ± 0.09*	1.61 ± 0.46*	0.11 ± 0.03	0.16
5.5	21.24 ± 0.18*	2.21 ± 0.17*	0.12 ± 0.01	0.20
6.0	9.77 ± 0.61*	2.72 ± 0.60	0.39 ± 0.11	0.29
Control	8.12 ± 0.51	3.42 ± 0.46	0.74 ± 0.14	0.38

Measurements are presented as mean ± SD (n = 3).

*$p < 0.05$ (significant difference with respect to caprylic acid fractionation method of baboon serum that was done before optimisation (control).

Table S2. The effect of different caprylic acid concentrations on the purification of baboon IgG.

CA concentration	Protein concentration (mg/mL)	Albumin concentration (mg/mL)	Albumin/ Globulin ratio	Turbidity (A600 nm)
5%	3.92 ± 0.04*	2.72 ± 0.30	2.43 ± 0.94	0.14
6%	8.06 ± 0.05	0.50 ± 0.17*	0.07 ± 0.02	0.42
7%	11.23 ± 0.46*	0.91 ± 0.30*	0.09 ± 0.03	0.04
8%	4.81 ± 0.12*	0.40 ± 0.17*	0.09 ± 0.05	0.05
9%	5.79 ± 0.22*	3.12 ± 0.46	1.19 ± 0.30	0.22
10%	8.30 ± 0.87*	3.32 ± 0.52	0.66 ± 0.06	0.46
12%	6.52 ± 0.37*	3.62 ± 0.30	1.25 ± 0.08	0.50
15%	6.62 ± 0.09	3.72 ± 0.63	1.35 ± 0.48	0.62
Control	8.12 ± 0.51	3.42 ± 0.46	0.74 ± 0.14	0.38

Measurements are presented as mean ± SD (n = 3).

*$p < 0.05$ (significant difference with respect to caprylic acid fractionation method of baboon serum that was done before optimisation (control).

Table S3. The effect of different stirring intensities of sera on caprylic acid purification of baboon IgG.

Stirring intensity (rpm)	Protein concentration (mg/mL)	Albumin concentration (mg/mL)	Albumin/ Globulin ratio	Turbidity (A600 nm)
200 (mild stirring)	7.11 ± 0.17*	2.76 ± 0.40	0.64 ± 0.13	0.26
800 (moderate stirring)	13.81 ± 0.03*	0.82 ± 0.10*	0.06 ± 0.01	0.08
1200 (vigorous stirring)	15.57 ± 0.26*	0.70 ± 0.09*	0.05 ± 0.01	0.03
1500 (extremely vigorous)	4.77 ± 0.15*	2.72 ± 0.30	1.38 ± 0.44	0.10
Control	8.12 ± 0.51	3.42 ± 0.46	0.74 ± 0.14	0.38

Measurements are presented as mean ± SD (n = 3).

*$p < 0.05$ (significant difference with respect to caprylic acid fractionation method of baboon serum that was done before optimisation (control).

Table S4. The effect of different stirring times on caprylic acid fractionation of baboon antisera.

Stirring time (min)	Protein concentration (mg/mL)	Albumin concentration (mg/mL)	Albumin/ Globulin ratio	Turbidity (A600 nm)
30	11.38 ± 0.01*	2.92 ± 0.35	0.35 ± 0.06	0.05
60	12.41 ± 0.01*	0.60 ± 0.03*	0.05 ± 0.00	0.05
90	12.22 ± 0.36*	0.94 ± 0.00*	0.08 ± 0.00	0.05
120	14.31 ± 0.04*	1.27 ± 0.11*	0.10 ± 0.01	0.05
Control	8.12 ± 0.51	3.42 ± 0.46	0.74 ± 0.14	0.38

Measurements are presented as mean ± SD (n = 3).

*$p < 0.05$ (significant difference with respect to caprylic acid fractionation method of baboon serum that was done before optimisation (control).

Table S5. The effect of different dialysis buffers on caprylic acid fractionation of baboon antisera.

Dialysis buffer	Protein concentration (mg/mL)	Albumin concentration (mg/mL)	Albumin/ Globulin ratio	Turbidity (A600 nm)
PBS	19.46 ± 1.06*	0.50 ± 0.02*	0.00 ± 0.00	0.03
SCS	20.54 ± 1.85*	0.74 ± 0.03*	0.00 ± 0.00	0.04
SPB	24.87 ± 0.24*	0.52 ± 0.04*	0.00 ± 0.00	0.05
Control	8.12 ± 0.51	3.42 ± 0.46	0.74 ± 0.14	0.38

Measurements are presented as mean ± SD (n = 3).

*$p < 0.05$ (significant difference with respect to caprylic acid fractionation method of baboon serum that was done before optimisation (control).

Table S6. The effect of lyophilisation on the final IgG product purified using caprylic acid.

Conditions	Protein concentration (mg/mL)	Albumin concentration (mg/mL)	Albumin/ Globulin ratio	Turbidity (A600 nm)
Before lyophilisation	24.87 ± 0.24*	0.74 ± 0.03*	0.03 ± 0.00	0.15
After lyophilisation	64.23 ± 1.46*	1.09 ± 0.20*	0.02 ± 0.00	0.10
Control	8.12 ± 0.51	3.42 ± 0.46	0.74 ± 0.14	0.38

Measurements are presented as mean ± SD (n = 3).

*$p < 0.05$ (significant difference with respect to caprylic acid fractionation method of baboon serum that was done before optimisation (control).

Table S7. Comparison of the purified baboon IgG before and after optimisation of the caprylic acid purification protocol.

Parameter	Pre-lyophilisation		Post-lyophilisation	
	Before optimisation	After optimisation	Before optimisation	After optimisation
Protein concentration (mg/ml)	8.12 ± 0.51	24.87 ± 0.24*	27.93 ± 1.21*	64.23 ± 1.46*
Albumin concentration (mg/mL)	3.42 ± 0.46	0.74 ± 0.03*	4.23 ± 0.80	1.09 ± 0.20*
Albumin/ Globulin ratio	0.74 ± 0.14	0.03 ± 0.00	0.18 ± 0.03	0.02 ± 0.00
Turbidity ($A_{600\ nm}$)	0.38	0.05	0.15	0.10

Measurements are presented as mean ± SD (n = 3).

*$p < 0.05$ (significant difference with respect to caprylic acid fractionation method of baboon serum that was done before optimisation (control).

Table S8. Comparison of caprylic acid purified baboon IgG with commercial IgG products.

Product	Protein concentration (mg/mL)	Albumin concentration (mg/mL)	Albumin/ Globulin ratio	Turbidity (A600 nm)
Equine IgG	100.46 ± 0.17*	0.85 ± 0.13	0.01 ± 0.00	0.02
CA-purified baboon IgG	64.23 ± 1.46	1.09 ± 0.20	0.02 ± 0.00	0.10
EchiTAb-plus-ICP	76.17 ± 1.16*	1.61 ± 0.17	0.02 ± 0.00	0.02

Measurements are presented as mean ± SD (n = 3).

*$p < 0.05$ (A significant difference with respect to caprylic acid fractionation method of baboon serum that was done after optimisation).
